# Prevalence of Antibodies to *Toxoplasma gondii* in Different Wild Bird Species Admitted to Rehabilitation Centres in Portugal

**DOI:** 10.3390/pathogens10091144

**Published:** 2021-09-05

**Authors:** Carolina Lopes, Ricardo Brandão, Ana Filipa Lopes, Roberto Sargo, María Casero, Carolina Nunes, Filipe Silva, Jitender P. Dubey, Luís Cardoso, Ana Patrícia Lopes

**Affiliations:** 1 Department of Veterinary Sciences, School of Agrarian and Veterinary Sciences (ECAV), University of Trás-os-Montes e Alto Douro (UTAD), 5000-801 Vila Real, Portugal; carolina3lopes@gmail.com (C.L.); rsargo@utad.pt (R.S.); fsilva@utad.pt (F.S.); aplopes@utad.pt (A.P.L.); 2 Centre for Ecology, Recovery and Surveillance of Wild Animals (CERVAS), Av. Bombeiros Voluntários 8, 6290-520 Gouveia, Portugal; brandaoric@gmail.com; 3 Wildlife Study and Rehabilitation Centre (CERAS), Quercus ANCN, Rua Tenente Valadim, 17, 6000-284 Castelo Branco, Portugal; anafilipa.sl@gmail.com; 4 Wildlife Rehabilitation Centre of the Veterinary Teaching Hospital of UTAD (CRAS-UTAD), Quinta de Prados, 5000-801 Vila Real, Portugal; 5 Wildlife Rehabilitation and Research Centre of Ria Formosa (RIAS), Parque Natural da Ria Formosa, 8700-194 Olhão, Portugal; mariavmcasero@gmail.com; 6 Wildlife Rehabilitation Centre of Santo André (CRASSA), Quercus ANCN, Moinho Novo, Galiza, 7500-022 Vila Nova de Santo André, Portugal; cnunes94@gmail.com; 7 Animal and Veterinary Research Centre (CECAV), University of Trás-os-Montes e Alto Douro (UTAD), 5000-801 Vila Real, Portugal; 8 Animal Parasitic Diseases Laboratory, Beltsville Agricultural Research Center, Agricultural Research Service, US Department of Agriculture, Beltsville, MD 20705, USA; jitender.dubey@usda.gov

**Keywords:** MAT, One Health, Portugal, seroprevalence, *Toxoplasma gondii*, wild birds, zoonosis

## Abstract

*Toxoplasma gondii* is a worldwide zoonotic parasite. According to the “One Health” approach, studies on toxoplasmosis are essential since it affects humans and domestic and wild animals. In the present study, antibodies to *T. gondii* were determined in serum samples from 263 wild birds located in five wildlife rehabilitation centres in mainland Portugal by using the modified agglutination test (MAT) with a cut-off titre of 20. An overall seroprevalence of 36.5% (95% confidence interval [CI]: 30.7–42.6) was observed. For the first time, antibodies to *T. gondii* were detected in some avian species, including pallid swift (*Apus pallidus*) (33.3%), black-backed gull (*Larus fuscus*) (39.3%), European turtle-dove (*Streptopelia turtur*) (100%), bee-eater (*Merops apiaster*) (50.0%), carrion crow (*Corvus corone*) (33.3%), and Egyptian vulture (*Neophron percnopterus*) (100%), which expands the list of intermediate hosts of *T. gondii*. A lower seroprevalence was found in juvenile birds (31.9%) compared to adults (48.7%) (*p* = 0.016). The central region of Portugal was considered a risk factor for *T. gondii* infection in wild birds (odds ratio: 3.61; 95% CI: 1.09–11.91). This pioneer study calls attention to the need for further studies, to provide a clearer understanding of *T. gondii* epidemiology in Portugal, because it reflects wide dispersion of *T. gondii* oocysts in the environment.

## 1. Introduction

*Toxoplasma gondii* is one of the most widespread zoonotic pathogens and its importance has increased in light of the “One Health” approach [1]. This protozoan may virtually affect all warm-blooded animals, i.e., mammals and birds; however, only members of family Felidae can act as definitive hosts [2].

The global decrease in the number of some avian species [3], together with the development of anthropogenic activities and the destruction of habitats, contributes to the interaction between the domestic and sylvatic cycles, reinforcing adjustments in the epidemiology of *T. gondii* [4,5]. Some species of birds develop clinical toxoplasmosis, which can be a considerable concern from the wildlife conservation perspective [6]. For instance, toxoplasmosis was reported in the Hawaiian crow or ‘alalã (*Corvus hawaiiensis*) and appears to pose a significant threat and a management challenge to the reintroduction programs of this endangered species [7].

Wild birds are particularly important intermediate hosts of *T. gondii* due to their high dispersal capabilities [6]. Along the flight path, migratory birds can carry infectious disease agents across oceans [8]. In addition, wild and domestic birds are excellent sentinels of environmental contamination with *T. gondii* oocysts, since herbivorous birds feed from the ground, and birds of prey eat intermediate hosts of *T. gondii* [9,10]. Consequently, there is a huge variety of wild bird species, with different habitats and diets, that may become infected with this parasite [6,11]. In the terrestrial environment, *T. gondii* prevalence increases with trophic level, consistent with predominant tissue cyst transmission, but in aquatic environment it reflects a considerable waterborne exposure to oocysts [6]. Birds of prey are predominantly infected by the consumption of small animals that have cysts in their tissues, but they also may become infected by drinking water contaminated with sporulated oocysts of *T. gondii* [12]. Some opportunistic birds, like seagulls, are also good indicators of environmental contamination, because they use dumps and sewers to feed themselves [9,13]. Additionally, there are game birds that may be consumed by humans and pose a risk of *T. gondii* transmission to them, if meat is not properly cooked [14,15].

Even though several studies have focused on the prevalence of *T. gondii* in wild birds worldwide [6,11], little information is available from Portugal. A previous study performed in northern and central regions of Portugal reported a high seroprevalence in wild mammals and birds [16]. The purpose of the present study was to provide updated information on the seroprevalence of *T. gondii* infection in wild birds admitted to rehabilitation centres across mainland Portugal. Furthermore, a risk factor analysis was performed and potential implications for the public health discussed.

## 2. Results

Antibodies to *T. gondii* were detected in 96 out of 263 birds, with an overall seroprevalence of 36.5% (95% confidence interval [CI]: 30.7–42.6). Most birds had low MAT titres: 20 in 78 birds, 400 in 7, 1600 in 8, and 6400 in 3 (Table 1).

Table 2 presents the seroprevalence of *T. gondii* infection in wild birds admitted to rehabilitation centres across Portugal according to the variables studied. By rehabilitation centre, 59.0% of the birds were seropositive at CRAS-UTAD (Wildlife Rehabilitation Centre of the Veterinary Teaching Hospital of UTAD), 32.9% at CERVAS (Centre for Ecology, Recovery and Surveillance of Wild Animals), 31.0% at CERAS (Wildlife Study and Rehabilitation Centre), 16.7% at CRASSA (Wildlife Rehabilitation Centre of Santo André), and 40.0% at RIAS (Wildlife Rehabilitation and Research Centre of Ria Formosa). A pairwise analysis (n = 10) revealed a difference between CRAS-UTAD and CRASSA (*p* = 0.007).

Regarding age, a significantly different seroprevalence was observed between juvenile (31.9%) and adult (48.7%) (*p* = 0.016) wild birds.

Significant differences (*p* = 0.005) were also observed for the geographical regions with a seroprevalence of 53.8% in the North, 36.2% in the Centre, 66.7% in Lisbon, 16.7% in Alentejo, and 41.3% in Algarve. Pairwise analysis revealed a significant difference between North and Alentejo (*p* = 0.001), between Centre and Alentejo (*p* = 0.021), and between Algarve and Alentejo (*p* = 0.016). 

For the four sample collection seasons compared, all together differences were not significant, although *p* value was very close to 0.05 (*p* = 0.053). Nevertheless, a paired analysis revealed a *p* value of 0.015 when comparing summer and winter.

For the remaining variables under study (i.e., gender, migratory behaviour, diet, and cause of entry), no statistically significant differences were found.

Geographical region was the only confirmed risk factor. Birds from central Portugal had an odds ratio (OR) of 3.61 (95% CI: 1.09–11.91; *p* = 0.035) when compared to the reference category (Alentejo).

## 3. Discussion

In the present study, antibodies to *T. gondii* were detected in 36.5% of 263 wild birds. A previous study by using the MAT and the same cut-off [16] reported 50.0% seroprevalence in wild birds in Portugal. Compared with our studies from Portugal, a Spanish study obtained a lower value of 26.1% by using a MAT cut-off value of 25 [17]. Several factors might explain the differences observed in the overall seroprevalence, but age of birds and the geographical region where they were found seem to be especially important [16,17,18,19]. 

Although some species were represented by less than 10 individuals, a circumstance which may limit representativeness, this study can be considered the most complete to date performed in wild birds in Portugal. Furthermore, to the best of our knowledge, this was the first report of antibodies to *T. gondii* in pale swift, lesser black-backed gull, European turtledove, bee-eater, carrion crow, and Egyptian vulture. These results add to the existing data on *T. gondii* infection in wild birds worldwide, as they add new bird species to the list of possible intermediate hosts.

In the present study, even though only one Egyptian vulture was tested, a titre of 20 was obtained, which may be an important finding, since it is an endangered species [20]. Interestingly, in Spain, 49 Egyptian vultures were tested but no antibodies to *T. gondii* were detected [17]. Several factors may explain these differences, including the cut-off used. Nevertheless, it is crucial to carry out additional studies.

The pale swift is a bird that spends most of its time in flight [21]. It normally hunts at high altitudes, feeding on flying arthropods whose dimensions increase with the age of the bird [22]. In the present report, a seroprevalence of 33.3% (95% CI: 4.3–77.7) in association with the behavioural characteristics of the pale swift suggested that *T. gondii* may be present in arthropods, which constitute the basis of pale swift diet or in drinking water. Studies indicate that *T. gondii* dispersion can occur through earthworms, flies, and cockroaches [2]. In fact, *T. gondii* oocysts were detected on the body surface of beetles (*Onthophagus* spp.) and remained infective for several months [23]. Moreover, *T. gondii* was detected in the digestive system of cockroaches (*Leucophaea maderae* and *Periplaneta americana*) [24]. Thus, the detection of *T. gondii* in pale swifts highlights the importance of considering alternative transmission routes for the parasite, namely by arthropods and drinking water.

The Eurasian buzzard is a bird with approximately 75% of its population distributed in Europe [20]. Consequently, it is a species frequently observed by humans and present in rehabilitation centres, making it a strong candidate for conducting epidemiological studies among different regions. Interestingly, seroprevalence values for *T. gondii* are quite variable in this species. For example, in the present study a figure of 47.8% was observed. Higher values were reported with 69.2% in the North and Centre of Portugal [16], 79% in France [25], 8.3% in Italy [26], and 51.0% in Spain [17]. These results probably reflect a considerable degree of infection in the small intermediate hosts that constitute the food source for birds of prey [16].

Despite the presence of high antibody titres in some birds, none of them appeared sick. Higher titres may not be associated with the severity of clinical signs, as a high titre may persist for months after infection [2]. Clinical toxoplasmosis has been observed in several avian species in the wild, but the most severe infections have occurred in captive canaries and finches [2]. The clinical signs are non-specific and may include anorexia, apathy, decreased body condition, diarrhoea, feather degradation, and dyspnoea [27]. Toxoplasmosis is thought to be related to retinal changes, especially in owls (*Strix aluco*) [28]. 

The seroprevalence of antibodies to *T. gondii* was significantly higher in adult birds (48.7%) compared to juveniles (31.9%) (*p* = 0.016). Similar results were found in the North and Centre regions of Portugal [16]. However, in the present study, only 28.9% of animals were adults. Therefore, the low percentage of adult individuals may have contributed to a lower overall seroprevalence, since adult birds are more likely to contact with infective stages of *T. gondii* throughout their lives. In Spain, birds over 1 year of age had a 6.8 times higher risk of being seropositive than the younger ones (95% CI: 2.71–17.29) [17]. Several reports around the world have found an increase in seroprevalence with age in wild birds [10,16,17,29,30]. Most birds are thought to acquire *T. gondii* infection after hatching, because *in ovo* transmission is rare [2]. In this regard, the most convincing data were provided by a study of migratory and non-migratory geese [8]. From a total 2675 birds, both adults and juveniles, only adults geese were seropositive [8]. 

In the present study, seroprevalence values were significantly different among geographical regions (53.8% in the North, 36.2% in the Centre, 66.7% in Lisbon, 16.7% in Alentejo, and 41.3% in Algarve). Birds from the central region of Portugal had a 3.61 higher risk of acquiring infection in comparison with birds from Alentejo (95% CI: 1.09–11.91). Variations between different regions of Portugal were also verified in cats and humans, although no region was identified as a risk factor. Regarding cats, seroprevalence was higher in Lisbon [31] compared to the North [32]. Concerning human infection, the seroprevalence values were higher in the North than in the south [33]. Most of the reports on *T. gondii* in Portugal are from the North and Lisbon, however, the present study emphasizes the importance of studying all regions of Portugal, to fill the gap of existing data, particularly in the centre. The seroprevalence values found in different regions may be associated with the physicochemical characteristics of the soil, humidity, temperature, and the presence of vegetation, since these factors can influence the persistence of oocysts in a given location [18]. For instance, in European countries, the prevalence of *T. gondii* infection is very low in cold climatic conditions and higher in warm climatic conditions [19]. In Portugal, there is also a considerable seroprevalence of *T. gondii* in humans [33,34], domestic animals [35], slaughtered pigs [35], and chickens [36]. Regarding wild animals, infection of *T. gondii* was identified not only in captivity [37], but also in free-living animals [16,38]. Likewise, the seroprevalence of this parasite in wild and domestic herbivores suggests a high contamination of the environment in Portugal [33].

Although the results were not considered statistically significant, seroprevalence was higher in spring (50.0%) and winter (58.3%). Variations in seroprevalence according to season may not only be associated with weather conditions, but also with disposable food [39]. 

Regarding gender, no statistically significant differences were found between males and females, which suggests that both genders of wild birds are equally exposed and susceptible to *T. gondii* infection. This may be possibly because males and females share the same eating habits [30], although comparisons are difficult to obtain due to wild bird sexing difficulties [16]. This result agrees with other studies carried out in wild birds [16,30].

In the present study, the seroprevalence in resident birds (non-migratory) (39.5%) was slightly higher than the other birds, but differences were not significant. In Spain, sedentary birds had significantly higher seroprevalence (31.1%) compared to migratory birds (12.0%) [17]. It is possible that migratory birds have fewer opportunities to be exposed to the parasite, because migratory birds and mammals have faster paces of life than their non-migratory relatives [40]. Even so, the detection of antibodies to *T. gondii* in migratory birds assumes special importance, as they can transport the parasite over long distances [8]. 

It was expected that carnivorous birds presented higher seroprevalence values [6,17,41]. However, in the present report this was not verified, possibly due to the reduced number of granivore and insectivore birds, in comparation to carnivores. Furthermore, for the same category there are different habits of eating. For example, some carnivorous are scavengers and others are predators, with studies having shown that the seroprevalence in scavengers birds is lower than in birds of prey [6,17]. The prevalence of *T. gondii* infection increases with the trophic level, especially in terrestrial environments [6]. Top predators, such as the Eurasian eagle-owl, tend to have higher seroprevalence values [17]. In general, carnivorous birds are mainly infected by consuming prey with cysts in their tissues; however, they can also become infected from sporulated oocysts, e.g. by drinking contaminated water [12].

Infection in seagulls and other birds that take advantage of sewers and dumps are good indirect indicators of environmental contamination with oocysts [9,13]. In the present study, a further degree of a potential environmental contamination may be inferred by analysing species such as the white stork (31.4%), heron (100%), lesser black-backed gull (39.3%), and yellow-legged gull (43.5%). Generally, these birds feed by capturing fish, frogs, insects, small mammals, birds, or reptiles, and make use of food discarded by humans, and because of that, end up moving closer to the urban environments [13,42,43]. Changes in biodiversity can lead to significant differences in the type and amount of food available to birds, which can impact the epidemiology of *T. gondii* [10]. Considering that these are increasingly urban species, the recorded seroprevalence values are useful to warn about the environmental contamination to which human beings are subject.

Regarding the cause of entry of the birds in wildlife rehabilitation centres, the ones that fell from the nest, became orphans or were disoriented juveniles had a seroprevalence of 26.3%, which is a lower value in comparison with other causes. Although these differences were not statistically significant, this fact can be explained by the increasing seroprevalence in adults, which was also observed in this study.

In birds that were admitted to the rehabilitation centres due to trauma, the seroprevalence of antibodies to *T. gondii* was 37.8%, but it is not possible to prove a statistical association. Nevertheless, samples obtained from road-killed animals in Tasmania had significantly higher seroprevalence of *T. gondii* than those obtained from culled individuals, suggesting there may be behavioural differences associated with infection [44]. However, other studies did not find evidence that parasitized birds were more likely to be killed in road accidents [39]. Furthermore, a study carried out in Taiwan showed that clinical abnormalities, including bone fractures, animal bites, malnutrition, and weakness, were associated with *T. gondii* infection in wild birds [29]. Birds infected with *T. gondii* may respond more slowly to predators or other obstacles, becoming more vulnerable for caught or injury [29]. Also, humans with chronic *T. gondii* infection have longer reaction times and a greater risk of suffering car accidents [45].

Data were obtained only from birds admitted to rehabilitation centres, which may be a limitation, since results reflect the seroprevalence of *T. gondii* in birds that were confined. Of the 20 birds analysed showing weakness, it was possible to detect antibodies to *T. gondii* in 35.0%. Birds rarely have clinical toxoplasmosis [27], but it is possible that, similarly to what happens in humans [46], immunocompromised or debilitated birds may became sick. A lower seroprevalence was found in healthy birds (11%) than in sick birds (50%) [30]. Additional studies are needed to establish a relationship between the infection with *T. gondii* and the disease in birds.

## 4. Materials and Methods

Various rehabilitation centres, located in different cities from north to south of Portugal, contributed to this study. Namely, from the North, in Vila Real (Wildlife Rehabilitation Centre of the Veterinary Teaching Hospital of UTAD [CRAS-UTAD]); from the Centre, in Gouveia (Centre for Ecology, Recovery and Surveillance of Wild Animals [CERVAS]) and in Castelo Branco (Wildlife Study and Rehabilitation Centre [CERAS]); from Alentejo, in Santiago do Cacém (Wildlife Rehabilitation Centre of Santo André [CRASSA]); and from Algarve, in Olhão (Wildlife Rehabilitation and Research Centre of Ria Formosa [RIAS]).

Blood samples were collected between July 2019 and December 2020, according to the casuistry of each centre. Each year, the centres received a different number of wild animals: CRAS-UTAD received between 300 and 400; CERVAS between 500 and 600; CERAS between 300 and 400; CRASSA between 100 and 300; and RIAS between 3000 and 4000. In all centres, birds are the main species received.

Samples were collected from 263 wild birds admitted in rehabilitation centres due to variable causes and with no particularity regarding acceptance. The collection technique and the amount of blood collected were suitable for each bird, according to the species, clinical status, physical condition, and size. Blood was taken from the brachial or metatarsal vein or intracardially (when euthanasia was preformed due to the poor clinical condition of the animal). Blood was then placed into sterile tubes without an anti-coagulant. After clotting, at room temperature (for a maximum period of 24 hours), serum was separated and stored at −20 °C. In the cases when the clot did not form well, the samples were centrifuged (2000 rpm for 10 minutes) to amplify the quantity of serum. For each sample, information was registered regarding identification of the rescue centre where the sample was collected, scientific name, order, location, clinical history, sex, age, diet, migratory habits, and season.

Samples were later transported and analysed by the MAT at the Laboratory of Parasitology of the University of Trás-os-Montes e Alto Douro (UTAD), using a commercial kit (Toxo-Screen DA^®^, bioMérieux, Lyon, France), following the manufacturer´s instructions. Negative and positive controls were included in each test. A cut-off of 20 was chosen to maximize both sensitivity and specificity of the test [16,47,48], and to allow a viable comparison with the single study with data on the seroprevalence of *T. gondii* in wild birds in Portugal [16]. Serum samples were also tested at the dilutions of 1:400, 1:1600, and 1:6400. Several studies point to MAT as the most sensitive and specific, and it is a simple method to perform and widely available [27]. In addition, it has shown effectiveness in several species of wild birds [11,17], including in Portugal [16].

The birds were grouped into juveniles, adults or undetermined (in cases where their age was not known). The juvenile group also included chicks and sub-adults. Age assessment was carried out empirically by analysing the biometric values and plumage of each bird [49,50]. Regarding the geographical region of birds, mainland Portugal was divided according to the European Union Nomenclature of Units for Territorial Statistics (NUTS) II, namely North, Centre, Alentejo, Lisbon, and the Algarve regions. Additionally, sampled animals were grouped according to the season of sera collection: spring (March 21 to June 20), summer (June 21 to September 20), autumn (September 21 to June 20) and winter (December 21 to March 20). 

Birds were also grouped by sex (females, males, and undetermined). It was not possible to know the sex of all birds, as some did not show evident sexual dimorphism or were too young. Regarding type of diet, birds were grouped into granivorous, insectivorous, omnivorous, and carnivorous. Concerning the cause of entry in the rehabilitation centre, birds were subdivided into five categories (fall from the nest; trauma; intoxication; debility; and others). We were not able to determine which specific toxic was at the origin of the problem, in most cases of birds that entered with clinical signs compatible with cases of intoxication. The “debility” category included birds with low body condition, fragile and/or with difficulty standing up, and without any other apparent cause. Finally, “others” grouped, for example, punctual cases of birds from illegal captivity, defiled birds, and vestibular syndrome.

The chi-square or Fisher exact tests were used to compare seroprevalence values. Independent variables with significant difference between categories (probability [*p*] value < 0.05) were selected for multiple logistic regression analysis, in order to identify independent risk factors for seropositivity by calculating OR and their 95% CI [51]. Statistical analyses were done with IBMS SPSS Statistics 26.0^®^ software. Assuming a default 50% seroprevalence value, a 95% confidence level, and a 10% absolute error, 97 birds were calculated to include in this study [52]. A convenience sample of 263 birds that were submitted to clinical procedures corresponds to an absolute error of approximately 6.03%.

## 5. Conclusions

In conclusion, the present study support the scenario of a significant proportion of infected wild birds in Portugal, reflecting a wide dispersion of *T. gondii* oocysts in the environment and highlighting the importance of including wild birds in epidemiological studies of the parasite, since amplifying the list of intermediate hosts of *T. gondii*. This was a pioneer study and a call of attention to the need for further studies, in order to provide a clearer understanding of *T. gondii* epidemiology in Portugal.

## Figures and Tables

**Table 1:** Prevalence of *T. gondii* infection and antibody titres by species of wild birds from Portugal.

Order	Common Name (*Scientific Name*)	Number (%) Tested	Number (%) of MAT-Positive	95% CI	Antibody Titres (n)			
					20	400	1600	>6400
Accipitriformes	Northern goshawk (*Accipiter gentilis*)	7 (2.7)	3 (42.9)	9.9–81.6	3	0	0	0
	Eurasian sparrow hawk (*Accipiter nisus*)	7 (2.7)	2 (28.6)	3.7–71	2	0	0	0
	Cinereous vulture (*Aegypius monachus*)	6 (2.3)	1 (16.7)	0.4–64.1	1	0	0	0
	Booted eagle (*Aquila pennata*)	6 (2.3)	3 (50.0)	11.8–88.2	3	0	0	0
	Eurasian buzzard (*Buteo buteo*)	23 (8.7)	11 (47.8)	26.8–69.4	8	2	1	0
	Short-toed snake-eagle (*Circaetus gallicus*)	5 (1.9)	2 (40.0)	5.3–85.3	2	0	0	0
	Western marsh-harrier (*Circus aeruginosus*)	2 (0.8)	0 (0.0)	0.0–84.2	0	0	0	0
	Griffon vulture (*Gyps fulvus*)	21 (8.0)	3 (14.3)	3.1–36.3	3	0	0	0
	Black kite (*Milvus migrans*)	10 (3.8)	4 (40.0)	12.2–73.8	3	0	0	1
	Red kite (*Milvus milvus*)	7 (2.7)	2 (28.6)	3.7–71	2	0	0	0
	Egyptian vulture (*Neophron percnopterus*)	1 (0.4)	1 (100.0)	2.5–100	1	0	0	0
	European honey-buzzard (*Pernis apivorus*)	3 (1.1)	0 (0.0)	0.0–70.8	0	0	0	0
Apodiformes	Pallid swift (*Apus pallidus*)	6 (2.3)	2 (33.3)	4.3–77.7	2	0	0	0
Bucerotiformes	Common hoopoe (*Upupa epops*)	1 (0.4)	1 (100.0)	2.5–100	1	0	0	0
Caprimulgiformes	European nightjar (*Caprimulgus europaeus*)	8 (3.0)	4 (50.0)	15.7–84.3	4	0	0	0
Charadriiformes	Lesser black-backed gull (*Larus fuscus*)	28 (10.6)	11 (39.3)	21.5–59.4	5	2	4	0
	Yellow-legged gull (*Larus michahellis*)	23 (8.7)	10 (43.5)	23.2–65.5	6	1	2	1
Ciconiiformes	White stork (*Ciconia ciconia*)	35 (13.3)	11 (31.4)	16.9–49.3	9	1	0	1
Columbiformes	Rock dove (*Columba livia*)	1 (0.4)	0 (0.0)	0.0–97.5	0	0	0	0
	European turtle-dove (*Streptopelia turtur*)	1 (0.4)	1 (100.0)	2.5–100	1	0	0	0
Coraciiformes	European bee-eater (*Merops apiaster*)	2 (0.8)	1 (50.0)	1.3–98.7	1	0	0	0
Falconiformes	Peregrine falcon (*Falco peregrinus*)	2 (0.8)	1 (50.0)	1.3–98.7	0	0	1	0
	Common kestrel (*Falco tinnunculus*)	1 (0.4)	0 (0.0)	0.0–97.5	0	0	0	0
Passeriformes	Carrion crow (*Corvus corone*)	3 (1.1)	1 (33.3)	0.8–90.6	1	0	0	0
Pelecaniformes	Grey heron (*Ardea cinerea*)	3 (1.1)	3 (100.0)	29.2–100	3	0	0	0
*Strigiformes*	Short-eared owl (*Asio flammeus*)	2 (0.8)	1 (50.0)	1.3–98.7	1	0	0	0
	Northern long-eared owl (*Asio otus*)	1 (0.4)	1 (100.0)	2.5–100	1	0	0	0
	Little owl (*Athene noctua*)	12 (4.6)	2 (16.7)	2.1–100	2	0	0	0
	Eurasian eagle-owl (*Bubo bubo*)	2 (0.8)	1 (50.0)	1.3–98.7	1	0	0	0
	Tawny owl (*Strix aluco*)	15 (5.7)	6 (40.0)	16.3–67.7	6	0	0	0
	Common barn-owl (*Tyto alba*)	18 (6.8)	7 (38.9)	17.3–64.3	6	1	0	0
*Suliforme*	Northern gannet (*Morus bassanus*)	1 (0.4)	0 (0.0)	0.0–97.5	0	0	0	0
Total	263 (100)	96 (35.6)	30.7–42.6	78	7	8	3	

CI: confidence interval; MAT: Modified agglutination test. Information is presented by alphabetical order, both for the orders and scientific names of birds.

**Table 2:** Seroprevalence of *T. gondii* infection in wild birds admitted to rehabilitation centres across Portugal according to the variables studied.

Variable	Number (%) Tested	Number (%) of MAT-Positive	95% CI
Rehabilitation centre			
CRAS-UTAD	39 (14.8)	23 (59.0)	42.1–74.4
CERVAS	85 (32.3)	28 (32.9)	23.1–44.0
CERAS	71 (27.0)	22 (31.0)	20.5–43.1
CRASSA	18 (6.8)	3 (16.7)	3.6–41.4
RIAS	50 (19.0)	20 (40.0)	26.4–54.8
		*p* = 0.010	
Order			
Accipitriformes	97 (36.9)	31 (32.0)	22.9–42.2
Charadriiformes	51 (19.4)	21 (41.2)	27.6–55.8
Ciconiiformes	35 (13.3)	11 (31.4)	16.9–49.3
Strigiformes	51 (19.4)	19 (37.3)	24.1–51.9
Other ^a^	29 (11.0)	14 (48.3)	29.5–67.5
		*p =* 0.481	
Age			
Juvenile	182 (69.2)	58 (31.9)	25.2–39.2
Adult	76 (28.9)	37 (48.7)	37.0–60.4
Undetermined ^§^	5 (1.9)	1 (20.0)	0.5–71.6
		*p =* 0.016	
Geographical region			
North	39 (14.8)	21 (53.8)	37.2–69.9
Centre	127 (48.3)	46 (36.2)	27.9–45.2
Lisbon	3 (1.1)	2 (66.7)	9.4–99.2
Alentejo	48 (18.3)	8 (16.7)	7.5–30.2
Algarve	46 (17.5)	19 (41.3)	27.0–56.8
		*p =* 0.005	
Season			
Spring	6 (2.3)	3 (50.0)	11.8–88.2
Summer	111 (42.2)	33 (29.7)	21.4–39.2
Autumn	122 (46.4)	46 (37.7)	29.1–46.9
Winter	24 (9.1)	14 (58.3)	36.6–77.9
		*p* = 0.053	
Sex			
Female	26 (9.9)	7 (26.9)	11.6–47.8
Male	30 (11.4)	12 (40.0)	22.7–59.4
Undetermined ^§^	207 (78.7)	77 (37.2)	30.6–44.2
		*p* = 0.455	
Migratory behaviour			
Resident	81 (30.8)	32 (39.5)	28.8–51.0
Migratory	81 (30.8)	31 (38.3)	27.7–49.7
Mixed	101 (38.4)	33 (32.7)	23.7–42.7
		*p* = 0.588	
Diet			
Granivorous	2 (0.8)	1 (50.0)	1.3–98.7
Insectivorous	17 (6.5)	8 (47.1)	23.0–72.2
Omnivorous	54 (20.5)	22 (40.7)	27.6–55.0
Carnivorous	190 (72.2)	65 (34.2)	27.5–41.4
		*p =* 0.610	
Cause of entry			
Debility	20 (7.6)	7 (35.0)	15.4–59.2
Others	42 (16.0)	18 (42.9)	27.7–59.0
Intoxication	46 (17.5)	19 (41.3)	27.0–56.8
Fall from the nest	57 (21.7)	15 (26.3)	15.5–39.7
Trauma	98 (37.3)	37 (37.8)	28.2–48.1
		*p =* 0.430	
TOTAL	263 (100)	96 (36.5)	30.7–42.6

^a^ Other: Apodiformes, Bucerotiformes, Caprimulgiformes, Columbiformes, Coraciiformes, Falconiformes, Passeriformes, Pelecaniformes and Suliformes; ^§^ Not included in statistical analysis. MAT: modified agglutination test; CI: confidence interval.

## Data Availability

Data supporting the conclusions of this article are included within the article and its additional file. The datasets used and/or analysed during the present study are available from the corresponding author on reasonable request.

## References

[1] Aguirre A.A., Longcore T., Barbieri M., Dabritz H., Hill D., Klein P.N., Lepczyk C., Lilly E.L., McLeod R., Milcarsky J. (2019). The One Health approach to toxoplasmosis: Epidemiology, control, and prevention strategies. EcoHealth.

[2] Dubey J.P. (2010). Toxoplasmosis of Animals and Humans.

[3] Rosenberg K.V., Dokter A.M., Blancher P.J., Sauer J.R., Smith A.C., Smith P.A., Stanton J.C., Panjabi A., Helft L., Parr M. (2019). Decline of the North American avifauna. Science.

[4] Rêgo W.M.F., Costa J.G.L., Baraviera R.C.A., Pinto L.V., Bessa G.L., Lopes R.E.N., Silveira J.A.G., Vitor R.W.A. (2018). Genetic diversity of *Toxoplasma gondii* isolates obtained from free-living wild birds rescued in Southeastern Brazil. Int. J. Parasitol. Parasites Wildl..

[5] Shwab E.K., Saraf P., Zhu X.-Q., Zhou D.-H., McFerrin B.M., Ajzenberg D., Schares G., Hammond-Aryee K., van Helden P., Higgins S.A. (2018). Human impact on the diversity and virulence of the ubiquitous zoonotic parasite *Toxoplasma gondii*. Proc. Natl. Acad. Sci. USA.

[6] Wilson A.G., Lapen D.R., Mitchell G.W., Provencher J.F., Wilson S. (2020). Interaction of diet and habitat predicts *Toxoplasma gondii* infection rates in wild birds at a global scale. Glob. Ecol. Biogeogr..

[7] Work T.M., Massey J.G., Rideout B.A., Gardiner C.H., Ledig D.B., Kwok O.C.H., Dubey J.P. (2000). Fatal toxoplasmosis in free-ranging endangered ‘alala from Hawaii. J. Wildl. Dis..

[8] Sandström C.A.M., Buma A.G.J., Hoye B.J., Prop J., Jeugd H., van der Voslamber B., Madsen J., Loonen M.J.J.E. (2013). Latitudinal variability in the seroprevalence of antibodies against *Toxoplasma gondii* in non-migrant and Arctic migratory geese. Vet. Parasitol..

[9] Dubey J.P., Pena H.F.J., Cerqueira-Cézar C.K., Murata F.H.A., Kwok O.C.H., Yang Y.R., Gennari S.M., Su C. (2020). Epidemiologic significance of *Toxoplasma gondii* infections in chickens (*Gallus domesticus*): The past decade. Parasitology.

[10] Iemmi T., Vismarra A., Mangia C., Zanin R., Genchi M., Lanfranchi P., Kramer L.H., Formenti N., Ferrari N. (2020). *Toxoplasma gondii* in the Eurasian kestrel (*Falco tinnunculus*) in northern Italy. Parasit. Vectors.

[11] Dubey J.P., Murata F.H.A., Cerqueira-Cézar C.K., Kwok O.C.H., Su C. (2020). Epidemiologic significance of *Toxoplasma gondii* infections in turkeys, ducks, ratites and other wild birds: 2009–2020. Parasitology.

[12] Dubey J.P., Atkinson C.T., Thomas N.J., Hunter D.B. (2008). Toxoplasma. Parasitic Diseases of Wild Birds.

[13] Cabezón O., Cerdà-Cuéllar M., Morera V., García-Bocanegra I., González-Solís J., Napp S., Ribas M.P., Blanch-Lázaro B., Fernández-Aguilar X., Antilles N. (2016). *Toxoplasma gondii* infection in seagull chicks is related to the consumption of freshwater food resources. PLoS ONE.

[14] Skorpikova L., Reslova N., Lorencova A., Plhal R., Drimaj J., Kamler J., Slany M. (2018). Molecular detection of *Toxoplasma gondii* in feathered game intended for human consumption in the Czech Republic. Int. J. Food Microbiol..

[15] Zhang X.-X., Qin S.-Y., Li X., Ren W.-X., Hou G., Zhao Q., Ni H.-B. (2019). Seroprevalence and related factors of *Toxoplasma gondii* in pigeons intended for human consumption in northern China. Vector Borne Zoonotic Dis..

[16] Lopes A.P., Sargo R., Rodrigues M., Cardoso L. (2011). High seroprevalence of antibodies to *Toxoplasma gondii* in wild animals from Portugal. Parasitol. Res..

[17] Cabezón O., García-Bocanegra I., Molina-López R., Marco I., Blanco J.M., Höfle U., Margalida A., Bach-Raich E., Darwich L., Echeverría I. (2011). Seropositivity and risk factors associated with *Toxoplasma gondii* infection in wild birds from Spain. PLoS ONE.

[18] Shapiro K., Bahia-Oliveira L., Dixon B., Dumètre A., de Wit L.A., VanWormer E., Villena I. (2019). Environmental transmission of *Toxoplasma gondii*: Oocysts in water, soil and food. Food Waterborne Parasitol..

[19] Al-Malki E.S. (2021). Toxoplasmosis: Stages of the protozoan life cycle and risk assessment in humans and animals for an enhanced awareness and an improved socio-economic status. Saudi J. Biol. Sci..

[20] BirdLife International IUCN Red List for Birds. http://www.birdlife.org.

[21] Hedenström A., Norevik G., Boano G., Andersson A., Bäckman J., Åkesson S. (2019). Flight activity in pallid swifts *Apus pallidus* during the non-breeding period. J. Avian Biol..

[22] Cucco M., Bryant D.M., Malacarne G. (1993). Differences in diet of common (*Apus apus*) and pallid (*A. pallidus*) swifts. Avocetta.

[23] Saitoh Y., Itagaki H. (1990). Dung beetles, *Onthophagus spp*., as potential transport hosts of feline coccidia. Nihon Juigaku Zasshi.

[24] Wallace G.D. (1972). Experimental transmission of *Toxoplasma gondii* by cockroaches. J. Infect. Dis..

[25] Aubert D., Terrier M.-E., Dumètre A., Barrat J., Villena I. (2008). Prevalence of *Toxoplasma gondii* in raptors from France. J. Wildl. Dis..

[26] Gazzonis A.L., Zanzani S.A., Santoro A., Veronesi F., Olivieri E., Villa L., Lubian E., Lovati S., Bottura F., Epis S. (2018). *Toxoplasma gondii* infection in raptors from Italy: Seroepidemiology and risk factors analysis. Comp. Immunol. Microbiol. Infect. Dis..

[27] Dubey J.P. (2002). A review of toxoplasmosis in wild birds. Vet. Parasitol..

[28] Williams D.L., Gonzalez Villavincencio C.M., Wilson S. (2006). Chronic ocular lesions in tawny owls (*Strix aluco*) injured by road traffic. Vet. Rec..

[29] Chen J.-C., Tsai Y.-J., Wu Y.-L. (2015). Seroprevalence of *Toxoplasma gondii* antibodies in wild birds in Taiwan. Res. Vet. Sci..

[30] Naveed A., Ali S., Ahmed H., Simsek S., Rizwan M., Kaleem I., Gondal M.A., Shabbir A., Pervaiz F., Khan M.A. (2019). Seroprevalence and risk factors of *Toxoplasma gondii* in wild birds of Punjab Province, Pakistan. J. Wildl. Dis..

[31] Waap H., Cardoso R., Leitão A., Nunes T., Vilares A., Gargaté M.J., Meireles J., Cortes H., Ângelo H. (2012). In vitro isolation and seroprevalence of *Toxoplasma gondii* in stray cats and pigeons in Lisbon, Portugal. Vet. Parasitol..

[32] Lopes A.P., Cardoso L., Rodrigues M. (2008). Serological survey of *Toxoplasma gondii* infection in domestic cats from northeastern Portugal. Vet. Parasitol..

[33] Lopes A.P., Dubey J.P., Dardé M.-L., Cardoso L. (2014). Epidemiological review of *Toxoplasma gondii* infection in humans and animals in Portugal. Parasitology.

[34] Gargaté M.J., Ferreira I., Vilares A., Martins S., Cardoso C., Silva S., Nunes B., Gomes J.P. (2016). *Toxoplasma gondii* seroprevalence in the Portuguese population: Comparison of three cross-sectional studies spanning three decades. BMJ Open.

[35] Esteves F., Aguiar D., Rosado J., Costa M.L., de Sousa B., Antunes F., Matos O. (2014). *Toxoplasma gondii* prevalence in cats from Lisbon and in pigs from centre and south of Portugal. Vet. Parasitol..

[36] Rodrigues F.T., Moreira F.A., Coutinho T., Dubey J.P., Cardoso L., Lopes A.P. (2019). Antibodies to *Toxoplasma gondii* in slaughtered free-range and broiler chickens. Vet. Parasitol..

[37] Tidy A., Frangueiro S., Dubey J.P., Cardoso L., Lopes A.P. (2017). Seroepidemiology and risk assessment of *Toxoplasma gondii* infection in captive wild birds and mammals in two zoos in the North of Portugal. Vet. Parasitol..

[38] Waap H., Nunes T., Vaz Y., Leitão A. (2016). Serological survey of *Toxoplasma gondii* and *Besnoitia besnoiti* in a wildlife conservation area in southern Portugal. Vet. Parasitol. Reg. Stud. Rep..

[39] Lohr M.T., Lohr C.A., Burbidge A.H., Davis R.A. (2020). *Toxoplasma gondii* seropositivity across urban and agricultural landscapes in an Australian owl. Emu Austral. Ornithol..

[40] Soriano-Redondo A., Gutiérrez J.S., Hodgson D., Bearhop S. (2020). Migrant birds and mammals live faster than residents. Nat. Commun..

[41] Bártová E., Lukášová R., Vodička R., Váhala J., Pavlačík L., Budíková M., Sedlák K. (2018). Epizootological study on *Toxoplasma gondii* in zoo animals in the Czech Republic. Acta Trop..

[42] Pistorius P.A. (2008). Grey heron (*Ardea cinerea*) predation on the Aldabra white-throated rail (*Dryolimnas cuvieri aldabranus*). Wilson J. Ornithol..

[43] Peris S.J. (2003). Alimentación en basureros: La ingestión de objetos de plástico por la cigüeña blanca (*Ciconia ciconia*). Ardeola.

[44] Hollings T., Jones M., Mooney N., McCallum H. (2013). Wildlife disease ecology in changing landscapes: Mesopredator release and toxoplasmosis. Int. J. Parasitol. Parasites Wildl..

[45] Flegr J., Havlícek J., Kodym P., Malý M., Smahel Z. (2002). Increased risk of traffic accidents in subjects with latent toxoplasmosis: A retrospective case-control study. BMC Infect. Dis..

[46] Khan K., Khan W. (2018). Congenital toxoplasmosis: An overview of the neurological and ocular manifestations. Parasitol. Int..

[47] Amouei A., Sharif M., Hosseini S.A., Sarvi S., Mizani A., Salehi S., Gholami S., Jafar-Ramaji T., Daryani A. (2018). Prevalence of *Toxoplasma gondii* infection in domestic and migrating birds from Mazandaran province, Northern Iran. Avian Biol. Res..

[48] Huang S.-Y., Fan Y.-M., Chen K., Yao Q.-X., Yang B. (2019). Seroprevalence and risk assessment of *Toxoplasma gondii* in Java sparrows (*Lonchura oryzivora*) in China. BMC Vet. Res..

[49] Martínez J.A., Zuberogoitia I., Alonso R. (2002). Rapaces Nocturnas. Guía para la Determinación de la Edad y el Sexo en las Estrigiformes Ibéricas.

[50] Svensson L., Mullarney K., Zetterström D. (2017). Guia de Aves.

[51] Petrie A., Watson P. (2013). Statistics for Veterinary and Animal Science.

[52] Thrusfield M., Christley R. (2018). Veterinary Epidemiology.

